# Prediction of unerupted canines and premolars in a Syrian sample

**DOI:** 10.1186/2196-1042-15-4

**Published:** 2014-01-06

**Authors:** Ahmad S Burhan, Fehmieh R Nawaya

**Affiliations:** Department of Orthodontics, Faculty of Dentistry, Al-Baath University, Homs, Syria; Department of Pediatric Dentistry, Faculty of Dentistry, Syrian Private University, Damascus Countryside, Syria

**Keywords:** Prediction, Regression equation, Unerupted, Mixed dentition

## Abstract

**Background:**

The current study aimed to evaluate the applicability of the methods of Moyers and of Tanaka and Johnston to estimate the mesiodistal widths of permanent canines and premolars in Syrian individuals, to determine whether the predicting equations differ by sex, and to develop more accurate regression equations using various teeth groups as predictors.

**Methods:**

A total of 670 pretreatment pairs of casts belonging to 342 female patients and 328 male patients were selected from the archives of orthodontic clinics in Damascus University and Al-Baath University. All relevant teeth were measured to the nearest 0.01 mm. Paired *t* tests were used to test the applicability of the Moyers method and the Tanaka and Johnson equation on Syrian individuals. New regression equations were constructed.

**Results:**

The predicted values of permanent canines and premolars derived from Moyers' charts at the 50th percentile levels tended to underestimate the actual values for the male subjects but were comparable to the actual values for the female subjects. However, the predicted values derived at the 75th percentile levels tended to be comparable to the actual values for the male subjects and to overestimate the actual values for the female subjects. The predicted values calculated by Tanaka and Johnston's equations tended to overestimate the actual values in both study groups.

**Conclusions:**

The Moyers method was more accurate for the mixed dentition analysis for Syrian individuals. However, the proper percentile level is determined by sex. The use of the equations constructed using the Syrian sample is advised.

## Background

Orthodontic treatment in the mixed dentition is largely dependent on an accurate space analysis [1, 2]. This analysis is one of the important criteria in determining whether a treatment plan should involve serial extraction, guidance of eruption, space maintenance, space regaining, or merely periodic observation [3, 4].

Tooth size predictions of unerupted permanent canines and first and second premolars are critical aspects in mixed dentition space analysis. Three main approaches have been used to estimate the mesiodistal crown widths of permanent canines and premolars in mixed dentition patients:

1. Measurement of the unerupted teeth using radiographs [5–7],
2. Application of regression equations that relate the mesiodistal widths of erupted teeth to those of unerupted teeth [2, 8, 9],
3. A combination of measurements from erupted teeth and radiographs of unerupted teeth [10, 11].

Prediction methods based on the mesiodistal widths of erupted permanent teeth and/or dimensions of radiographic images of unerupted teeth usually employ simple or multiple linear regressions. The use of several predictors in multiple linear regressions may improve the prediction, but this approach may be overly complicated for clinical use. However, if an appropriate predictor is chosen for simple linear regression analysis, acceptable accuracy can still be obtained [12].

Moyers' analysis uses the sum of the widths of the mandibular incisors to predict the sum of both the mandibular and maxillary canines and premolars at various probability levels (5% to 95%), initially as combined tables for both sexes [1] and later as separate tables for either sex [2]. Neither the sample nor the regression equations, upon which Moyers' tables [1, 2, 8] are based, have been described in the literature. However, Moyers recommended its use at the 75% probability level, which is thought to err on the side of overestimating crowding. Despite the questionable reliability, both Moyers' [2] and the Tanaka and Johnston's [9] approaches are still widely accepted because they do not require radiographs and are simple and quick to perform. Thus, these methods are, arguably, more readily applied by a spectrum of clinicians [13]. Tanaka and Johnston [9] also used the sum of the mesiodistal widths of the mandibular central and lateral incisors to develop regression equations for predicting the sizes of the unerupted canines and premolars. They established that the mesiodistal widths at the 75th percentile level can be predicted by halving the width of the mandibular incisors and adding 10.5 mm for the mandibular teeth and 11.0 mm for the maxillary teeth. Of the methods commonly employed today, this approach is one of the quickest and easiest. However, the standard errors of the estimates for the correlations were rather high (0.86 mm for the maxillary and 0.85 mm for the mandibular teeth) [13].

Recently, several researchers have evaluated the applicability of Tanaka and Johnston's [9] and Moyers' [2] methods for different ethnic groups: Schirmer and Wiltshire [14] for black South Africans, Lee-Chan et al. [3] for Asian Americans, Jaroontham and Godfrey [15] for the Thai population, Nourallah et al. [16] for Syrians, Diagne et al. [17] for the Senegalese population, and Alessandri Bonetti et al. [18] for northern Italians. Only one such study has been conducted for Syrians, but it did not consider sex differences [16]. Many studies on various populations have found differences in the tooth sizes between male and female subjects [12, 19, 20]. Therefore, more studies are needed to evaluate the applicability of these two prediction methods for the Syrian population.

The study aims were (1) to evaluate the applicability of the methods of Moyers and of Tanaka and Johnston to estimate the mesiodistal widths of upper and lower permanent canines and premolars in Syrian individuals, (2) to determine whether the predicting equations differ by sex, and (3) to attempt to find more accurate regression equations for predicting the sizes of unerupted canines and premolars for the Syrian population using various tooth groups as predictors.

## Methods

**Figure 1:**
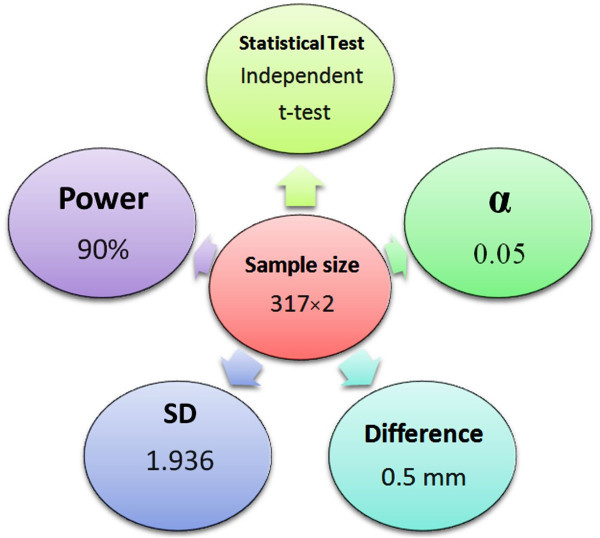
**Sample size estimation with its five assumptions.** Minitab software version 15 (Minitab Inc, State College, PA, USA) was used. *α*, significance level; SD, standard deviation.

The current research was designed as a cross-sectional analysis. A pilot study of 100 cases was performed to determine the proper sample size. The estimated value for the sample size is shown in Figure 1.

To select subjects, 1,542 pretreatment pairs of study casts derived from the archives of orthodontic clinics in Damascus University and Al-Baath University were examined. The accepted casts had to have the following criteria: belonging to Syrians with at least one previous generation of Syrian ancestors, belonging to patients 21 years old or younger, exhibiting full eruption for all permanent teeth in both arches (except third molars), lacking interproximal caries or restorations, lacking missing or supernumerary teeth, lacking abnormal-sized or abnormal-shaped teeth, lacking tooth wear, and being of high quality.

The final sample consisted of 670 patient casts (mean age 19.38 years, SD 1.66 years) derived from 328 male patients (mean age 19.42 years, SD 1.72 years) and 342 female patients (mean age 19.34 years, SD 1.63 years).

An electronic digital caliper (Gilbert, China) with an accuracy of 0.01 mm was used to measure the tooth sizes. To better adjust for interdental spaces, the measuring beaks were narrowed [21]. Only 10 casts were measured per session. The mesiodistal widths of teeth were recorded by only one examiner (ASB).

All teeth from the left molar through the right molar of each set of dental casts were measured to the nearest 0.01 mm. The caliper was held at the tooth's greatest mesiodistal diameter (anatomical contact points), parallel to the occlusal surface and perpendicular to the long axis of the tooth according to the method described by Seiple [22] and Moorrees et al. [23].

### Method error

To determine measurement reliability, 20 plaster casts were randomly selected and re-measured (first to first permanent molars, 480 tooth measurements), with intervals of at least 2 weeks. The measurement errors were statistically assessed using Dahlberg's formula [24].

### Statistical analysis

SPSS software (version 17, IBM, Chicago, IL, USA) was used to explore the data and design the tests. Kolmogorov-Smirnov tests were used to detect the normality distribution of the data. Paired *t* tests were performed to examine the bilateral symmetry of the mesiodistal widths of all measured individual teeth and other studied tooth groups in each arch. Independent *t* tests were performed to compare data between male and female subjects. Paired *t* tests were used to test the significance of the differences between the measured values and the predicted calculation using the Moyers and the Tanaka and Johnson methods. Pearson's coefficients were used to evaluate the correlations between the groups of teeth. Simple regression analyses of the dependent variables (the mean sum of the mesiodistal widths of the permanent canines and first and second premolars) were performed with the independent variables (which showed the highest Pearson's coefficients with the dependent variable) to develop possible regression equations for the Syrian sample. All the results were judged at the 95% confidence level.

## Results and discussion

### Results

The method errors showed that the differences between duplicate measurements ranged from 0.04 to 0.12 mm in both the mandibular and maxillary arches. The normalities of the distributions of groups of the right and left teeth and of the male and female tooth groups were examined using the Kolmogorov-Smirnov test. Because all the *p* values were greater than 0.05, the distributions were judged as normal.

The paired *t* test was used to compare between the right and left teeth. The results showed that there were no statistically significant differences between the two sides of the upper and lower arches in the total group.

The independent *t* test was used to compare the differences between male and female samples. Table 1 shows that there were statistically significant differences in the widths of teeth between the two groups. Male teeth were generally larger.

**Table 1:** **Comparisons of the mean differences in mesiodistal crown diameters (mm) between the male and female groups**

	Males		Females		***t*** value	***p*** value
	Mean	SD	Mean	SD		
Maxillary arch						
Central incisor	8.82	0.59	8.63	0.57	2.932	0.004
Lateral incisor	6.84	0.58	6.71	0.62	2.032	0.043
Canine	8.00	0.45	7.78	0.51	4.089	<0.001
First premolar	7.08	0.42	6.96	0.45	2.345	0.020
Second premolar	6.74	0.43	6.65	0.46	2.150	0.035
First molar	10.43	0.59	10.25	0.57	2.833	0.005
∑345	21.81	1.14	21.39	1.26	3.183	0.002
Mandibular arch						
Central incisor	5.56	0.41	5.47	0.41	2.0.35	0.040
Lateral incisor	6.12	0.42	5.98	0.44	2.871	0.004
Canine	7.01	0.48	6.73	0.46	5.469	<0.001
First premolar	7.17	0.45	7.04	0.49	2.452	0.015
Second premolar	7.27	0.44	7.15	0.47	2.405	0.017
First molar	11.31	0.57	11.07	0.64	3.527	<0.001
∑345	21.45	1.21	20.91	1.24	3.938	<0.001

∑345 is the sum of the permanent canines and premolars in one arch quarter.

The paired *t* test was used to compare the actual mesiodistal values of the sum of unerupted permanent canines, first and second premolars, and the predicted values calculated using Moyers' charts at the 50th and 75th percentile levels and using Tanaka and Johnston's method.

Table 2 shows that, in the male sample, the values predicted using Moyers' charts at the 50th percentile levels tended to underestimate the actual sum of the upper and lower permanent canines and premolars and the predicted values calculated by Tanaka and Johnston's equations tended to overestimate the actual sum of the upper and lower permanent canines and premolars. However, the predicted values derived from Moyers' charts at the 75th percentile levels were comparable with the actual values of the upper and lower permanent canines and premolars, lacking statistically significant differences.

**Table 2:** **Predicted values based on the two methods compared with the actual values in the male sample**

	Predicted values of ∑345		Actual values of ∑345		***t*** value	***p*** value
	Mean	SD	Mean	SD		
Maxillary arch						
Moyers 50%	21.55	1.17	21.81	1.14	−5.392	0.006
Moyers 75%	21.90	1.21	21.81	1.14	0.887	0.538
Tanaka and Johnston	22.17	1.79	21.81	1.14	6.578	0.003
Mandibular arch						
Moyers 50%	21.05	1.20	21.45	1.21	−6.639	0.004
Moyers 75%	21.55	1.21	21.45	1.21	0.952	0.475
Tanaka and Johnston	22.07	1.79	21.45	1.21	7.437	<0.001

∑345 is the sum of permanent canines and premolars in one arch quarter.

Table 3 shows that, in the female sample, predicted values derived from Moyers' charts at the 75th percentile levels and those calculated by Tanaka and Johnston's equations tended to overestimate the actual sum of the upper and lower permanent canines and premolars. However, the predicted values derived from Moyers' charts at the 50th percentile levels were comparable with the actual values of the upper and lower permanent canines and premolars without statistically significant differences.

**Table 3:** **Predicted values based on the two methods compared with the actual values in the female sample**

	Predicted values of ∑345		Actual values of ∑345		***t*** value	***p*** value
	Mean	SD	Mean	SD		
Maxillary arch						
Moyers 50%	21.55	1.17	21.39	1.26	1.123	0.231
Moyers 75%	21.90	1.21	21.39	1.26	6.874	<0.001
Tanaka and Johnston	22.17	1.79	21.39	1.26	8.987	<0.001
Mandibular arch						
Moyers 50%	21.05	1.20	20.91	1.24	1.112	0.365
Moyers 75%	21.55	1.21	20.91	1.24	7.455	<0.001
Tanaka and Johnston	22.07	1.79	20.91	1.24	9.583	<0.001

∑345 is the sum of permanent canines and premolars in one arch quarter.

Pearson's correlation coefficients were calculated between many independent variables and dependent variables. Table 4 shows that Pearson's correlation coefficients (*r*) were all above 0.5. Because the *r* values must be at least 0.7 (50% of the variance explained by the relationship between the two variables) to define a group of teeth as an accurate predictor, only predictors with *r* values of 0.7 or above were put into clinical orthodontic use by constructing regression equations for the Syrian sample.

**Table 4:** **Pearson's correlation coefficients (** ***r*** **) between each independent variable and dependent variable**

***X***	Males				Females			
	***Y*** = ∑345U		***Y*** = ∑345 L		***Y*** = ∑345U		***Y*** = ∑345 L	
	***r***	***p*** value	***r***	***p*** value	***r***	***p*** value	***r***	***p*** value
	0.588	<0.001	0.671	<0.001	0.707	<0.001	0.681	<0.001
	0.649	<0.001	0.709	<0.001	0.716	<0.001	0.745	<0.001
	0.646	<0.001	0.674	<0.001	0.712	<0.001	0.744	<0.001
	0.656	<0.001	0.734	<0.001	0.717	<0.001	0.755	<0.001
	0.676	<0.001	0.752	<0.001	0.748	<0.001	0.771	<0.001
	0.717	<0.001	0.776	<0.001	0.757	<0.001	0.781	<0.001
	0.716	<0.001	0.755	<0.001	0.755	<0.001	0.778	<0.001
	0.727	<0.001	0.780	<0.001	0.771	<0.001	0.799	<0.001

∑345 is the sum of the permanent canine and premolars in one arch quarter. U, upper; L, lower.

Generally, stronger correlations between each independent variable and dependent variable were noticed in the female group than in the male group and for the mandibular canines and premolars than the maxillary ones.

The regression characteristics of the obtained prediction equations for the Syrian sample are presented in Table 5 for the male subject group and in Table 6 for the female subject group. In this study, the standard error of the estimates (mean) ranged between 0.76 and 0.85 mm for the male group and 0.75 and 0.89 mm for the female group. The final regression equations derived from the Syrian sample are shown in Table 7.

**Table 5:** **Regression characteristics (** ***Y*** **=** ***BX*** **+** ***A*** **) for the male sample**

***X***	Y = ∑345U						***Y*** = ∑345 L			
	***r***	***r*** ^2^	Regression coefficient		Standard error of mean (mm)	***r***	***r*** ^2^	Regression coefficient		Standard error of mean (mm)
			***A***	***B***				***A***	***B***	
	-	-	-	-	-	-	-	-	-	-
	-	-	-	-	-	0.709	0.502	9.46	0.51	0.854
	-	-	-	-	-	-	-	-	-	-
	-	-	-	-	-	0.734	0.539	4.66	0.32	0.821
	-	-	-	-	-	0.752	0.565	4.38	0.37	0.798
	0.717	0.514	6.10	0.24	0.797	0.776	0.603	2.85	0.28	0.763
	0.716	0.512	2.97	0.25	0.798	0.755	0.570	2.10	0.26	0.793
	0.727	0.528	5.65	0.17	0.784	0.780	0.608	2.84	0.19	0.757

∑345 is the sum of permanent canines and premolars in one arch quarter. U, upper; L, lower.

**Table 6:** **Regression characteristics (** ***Y*** **=** ***BX*** **+** ***A*** **) for the female sample**

***X***	***Y*** = ∑345U						***Y*** = ∑345 L			
	***r***	***r*** ^2^	Regression coefficient		Standard error of mean (mm)	***r***	***r*** ^2^	Regression coefficient		Standard error of mean (mm)
			***A***	***B***				A	B	
	0.707	0.500	8.30	0.43	0.891	-	-	-	-	-
	0.716	0.512	8.76	0.55	0.879	0.745	0.555	7.74	0.58	0.833
	0.712	0.507	7.15	0.27	0.885	0.744	0.554	6.87	0.26	0.833
	0.717	0.515	4.28	0.33	0.878	0.755	0.570	4.00	0.33	0.819
	0.748	0.560	5.80	0.35	0.836	0.771	0.594	4.19	0.37	0.795
	0.757	0.573	4.32	0.26	0.823	0.781	0.610	2.50	0.28	0.780
	0.755	0.571	4.70	0.23	0.826	0.778	0.606	2.10	0.26	0.784
	0.771	0.594	3.39	0.19	0.802	0.799	0.639	2.43	0.19	0.750

∑345 is the sum of permanent canines and premolars in one arch quarter. U, upper; L, lower.

**Table 7:** **Syrian regression equations (** ***Y*** **=** ***BX*** **+** ***A*** **) for the male and female samples**

***X***	Males		Females	
	***Y*** = ∑345U	***Y*** = ∑345 L	***Y*** = ∑345U	***Y*** = ∑345 L
	*Y* = 0.17*X* + 5.65	*Y* = 0.19*X* + 2.84	*Y* = 0.19*X* + 3.39	*Y* = 0.19*X* + 2.43
	*Y* = 0.24*X* + 6.10	*Y* = 0.28*X* + 2.85	*Y* = 0.26*X* + 4.32	*Y* = 0.28*X* + 2.50
	*Y* = 0.25*X* + 2.97	*Y* = 0.26*X* + 2.10	*Y* = 0.23*X* + 4.70	*Y* = 0.26*X* + 2.10
	-	*Y* = 0.37*X* + 4.38	*Y* = 0.35*X* + 5.80	*Y* = 0.37*X* + 4.19
	-	*Y* = 0.32*X* + 4.66	*Y* = 0.33*X* + 4.28	*Y* = 0.33*X* + 4.00
	-	*Y* = 0.51*X* + 9.46	*Y* = 0.55*X* + 8.76	*Y* = 0.58*X* + 7.74
	-	-	*Y* = 0.27*X* + 7.15	*Y* = 0.26*X* + 6.87
	-	-	*Y* = 0.43*X* + 8.30	-

The equations are arranged in descending order according to Pearson's correlation coefficients between the dependent and independent variables. ∑345 is the sum of permanent canines and premolars in one arch quarter. U, upper; L, lower.

### Discussion

Measurement reliability, one of the most important aspects of odontometric studies, refers to the ability to obtain the same measurement consistently over sequential measures [25]. In an attempt to improve the reliability of the measurements studied herein, the following procedures were employed:

1. Use of high-quality dental casts made of dental stone [26],
2. Use of calipers with digital displays to greatly reduce eye fatigue and the possibility of reading error [26],
3. Assessing intra-examiner variability using Dahlberg's formula [24]. The method errors showed that differences between duplicate measurements ranged from 0.04 to 0.12 mm.

Therefore, any differences in the mesiodistal tooth widths, if observed, would result from the tooth size variability in the present sample and the prediction methods studied.

Because patients who seek treatment at orthodontic clinics at Damascus University and Al-Baath University come from all over the country, this sample was assumed to be representative of the Syrian population.

The determination of the sample size provided a sufficient power of 90% to determine any differences at a confidence interval of at least 95%. Therefore, this study has sufficiently many subjects for the clinically meaningful differences to be also statistically significant.

In this study, there were no statistically significant differences between the left and right sides. These findings indicate that the right or the left side measurements could be used to represent the mesiodistal tooth widths for this sample. This finding agreed with the usual practice of using teeth on one side of the jaw, or the average of the two, for analyzing the mesiodistal widths of teeth [4, 22, 23]. In this study, the averaged values of the right and left sides of each jaw were used in the statistical analyses [15, 23].

The results of independent *t* tests showed that there were statistically significant differences in the tooth widths between the male and female subjects. The mean mesiodistal tooth widths of male subjects were generally larger than those of females in both mandibular and maxillary dental arches (*p* < 0.05). Thus, data analysis was performed separately for each gender.

These results agree with many studies that have also found the average mesiodistal widths of individual male teeth to be larger than those of female teeth of permanent dentition in many ethnic groups [12, 19, 27].

In contrast, the mesiodistal measurements for the Iraqi males were generally larger than for the females; however, the difference was only statistically significant (*p* < 0.05) for the canines [20]. Other studies have found no significant differences in the mesiodistal widths of both maxillary and mandibular incisors [28].

Moyers' tables at the 50th percentile levels tended to underestimate the actual sum of permanent canine and premolars in the male sample, whereas the values were comparable with those of the female sample. Moyers' tables at the 75th percentile levels tended to be comparable with the actual sum of permanent canine and premolars in the male sample, whereas they tended to overestimate those values in the female sample. When Tanaka and Johnston's method was applied in the male and female samples, there was overestimation in both groups. The methods of Moyers and Tanaka and of Johnston were developed for North American individuals and have been tested in many other subjects from different origins [27–34].

Many studies have found that Moyers' tables at the 50th and 75th percentile levels tended to underestimate the actual sum of permanent canines and premolars [12]. Some authors found no differences when Moyers' method at 75th percentile was used [34]. In contrast, other studies found that these 75th percentile levels tended to overestimate the actual values [17].

The results of the Tanaka and Johnston method in the current study are in accordance with those of some other studies [3, 29, 35, 36]. However, other authors have found comparable estimation using this method [34]. The variability in the results found when the methods of Moyers and of Tanaka and Johnston were applied in Syrian individuals may be explained by the differences in the sample sizes and origins.

The current study attempted to examine Pearson's correlation coefficients (*r*) between the combined mesiodistal widths of the unerupted permanent canines and premolars and many other predictors, including the traditional predictors, lower incisors.

Many dental groups were selected for the prediction of unerupted permanent canine and premolars, which were the dependent variables. The selection was performed depending on the time of eruption; early eruption enables the values to be used as independent prediction variables from the early mixed stage of dentition.

Based on tests of the relations between the independent variables and dependent variables used by Moyers' charts and by Tanaka and Johnston's equations, there were statistically significant correlations between the widths of permanent canine and premolars and lower incisors in the maxilla (*r* = 0.65) and in the mandible (*r* = 0.71) in the male subject group. In the female subject group, the correlation coefficients of the same variables were also statistically significant in the maxilla (*r* = 0.72) and in the mandible (*r* = 0.75). This independent variable, which was used in Moyers' charts and Tanaka and Johnston's equations, does not present the highest correlation coefficients with the dependent variables. However, it is still more important in many cases because of its early eruption.

The previous findings called for new and more accurate equations based on the use of different tooth groups as predictors. Interestingly, Table 4 shows that Pearson's correlation coefficient increased with the number of teeth involved in the independent variables.

The highest correlation coefficients in both male and female groups were between the widths of permanent canines and premolars and the sum of maxillary and mandibular incisors and molars, which were in the maxilla (*r* = 0.73) and in the mandible (*r* = 0.78) in the male subject group as well as in the maxilla (*r* = 0.77) and in the mandible (*r* = 0.80) in the female subject group.

Most studies to date have found the sum of the four mandibular incisors to be one of best predictors in the linear regression equations for determining the combined mesiodistal widths of the unerupted permanent canines and premolars in both the mouth [37] and dental casts [2, 12]. Several clinical advantages of using the four permanent mandibular incisors in prediction equations and probability tables have previously been demonstrated [1, 9].

Many other studies tried to test the correlation values of other predictors [30–33, 38], finding acceptable Pearson's correlation coefficients (*r*) between the combined mesiodistal widths of the unerupted permanent canines and premolars and many predictors; however, their use in regressions is limited.

To change the statistical results to be clinically useful in the Syrian population, we had to develop regression equations based on the use of the various studied predictors. These regression equations are arranged in Table 7 in descending order according to Pearson's correlation coefficients between the dependent and independent variables. Thus, it is advisable to use the equations as they are arranged in the table in accordance with the erupted teeth.

## Conclusions

Tooth widths exhibit statistically significant differences between male and female subjects, with male teeth generally being larger. Thus, Syrian subjects should be divided according to gender prior to a mixed dentition analysis.

The Moyers method is more accurate for mixed dentition analysis for Syrian individuals. However, the proper percentile level is determined by sex. The predicted widths determined by the Tanaka and Johnston equations overestimate the actual widths of the lower permanent canines and premolars for male and female patients. The independent variable used in Moyers' charts and Tanaka and Johnston's equations does not exhibit the highest correlation coefficients with the dependent variables.

Regression equations based on many predictors were developed in the Syrian population. Validating studies must be conducted to confirm the applicability and precision of the new regression equations proposed.

## Authors’ information

ASB is a senior lecturer in the Department of Orthodontics, Faculty of Dentistry, Al-Baath University, Homs, Syria. FRN is a senior lecturer in the Department of Pediatric dentistry, Faculty of Dentistry, Syrian Private University, Damascus Countryside, Syria.
